# Racial disparities and in vitro fertilization (IVF) treatment outcomes: time to close the gap

**DOI:** 10.1186/s12958-020-00672-2

**Published:** 2020-11-19

**Authors:** Tarun Jain

**Affiliations:** 1 Division of Reproductive Endocrinology and Infertility, Department of Obstetrics and Gynecology, Northwestern University Feinberg School of Medicine, Chicago, IL USA; 2 Northwestern Center for Fertility and Reproductive Medicine, 676 North St Clair St, Suite 2310, Chicago, IL 60611 USA

From the first successful in vitro fertilization (IVF) cycle in 1978 through today, millions of deserving women and men have had the opportunity to build their families using this effective technology. The advent and advances of IVF treatment has truly been life changing. A major problem that has been identified in reproductive medicine, however, is that in the United States, access and outcomes to IVF are not equal. Black and Hispanic women are less likely than white women to access fertility care, and they are also less likely to have a successful IVF cycle [1].

Use of the Society for Assisted Reproductive Technology Clinical Outcomes Reporting System (SART CORS) national registry data has served as an effective research tool to identify such IVF outcome disparities [2]. The first such large registry-based study using IVF data from 1999 and 2000 found that the live-birth rate for black women was 18.7% compared to 26.3% for white women [3]. After controlling for confounding factors, the study found that black race was an independent risk factor for not achieving a live birth. Since that time, numerous additional studies have found similar results, including registry data analysis from 2004 to 2006.

Such findings led the American Society for Reproductive Medicine (ASRM) to make it a priority to address disparities and inequities in reproductive care. To see where we currently stand, Seifer and colleagues examined more recent SART CORS registry data from 2014 to 2016 [4]. They analyzed 122,721 autologous, fresh, non-donor IVF cycles, of which 13,717 cycles were from black women and 109,004 cycles were from white women. Not surprisingly, the investigators found that black women had a significantly lower live birth rate (OR 0.71) and a lower cumulative live birth rate (CLBR) for their initial cycle (OR 0.64), compared to white women (after adjusting for confounders). Interestingly, there was considerably less representation of cycles from black women compared to white women relative to their demographic representation in non-mandated compared to mandated states. Furthermore, the authors also found the CLBR was higher in mandated compared to non-mandated states for cycles from black women.

Unfortunately, black race continues to be an independent prognostic factor for live birth rate from IVF treatment. This major disparity seems to be further complicated by socioeconomic factors leading to disparate access and care among states with and without an insurance mandate to cover IVF treatment.

Such racial disparities are not unique to reproductive medicine and unfortunately widely pervade the United States healthcare system. There needs to be greater awareness and education surrounding such disparities, including assessment of our implicit biases. Furthermore, as noted by the Institute of Medicine (IOM) reports on disparities in healthcare, we all need to provide true patient-centered care and effective cross-cultural communication in order to improve quality, achieve equity, and ultimately eliminate the significant racial/ethnic disparities in health care that persist today.
